# Risk factors for implant-associated infection after bone allograft reconstruction: a 20-year retrospective cohort study

**DOI:** 10.1007/s00402-026-06431-z

**Published:** 2026-07-23

**Authors:** Fabrice Scheurer, Maria Smolle, Georg Hauer, Paul Ruckenstuhl, Thomas Valentin, Andreas Leithner

**Affiliations:** 1 Department of Orthopaedics, Universitätsklinik Balgrist, Zurich, Switzerland; 2 Department of Orthopaedics, Medical University of Graz, Graz, Austria; 3 Department of Infectiology, Medical University of Graz, Graz, Austria

**Keywords:** Bone allograft, Implant associated infection, Risk factors, Smoking, Body mass index, Radiotherapy

## Abstract

**Aims:**

Reconstruction of bone defects is a key challenge in orthopaedic surgery. While autologous bone grafts are considered the gold standard, osseous allografts are increasingly used as an alternative as they allow for treatment of large bone defects. However, they may be associated with an increased risk of postoperative infections. This study aims to analyze the frequency of and risk factors for implant-associated infections after bone allograft transplantation.

**Methods:**

All patients who underwent reconstruction with bone allografts at a single orthopaedic and trauma unit between 2003 and 2023 were retrospectively included. Data on demographics, disease-associated parameters, risk factors at index surgery, and development of implant-associated infections during follow-up were documented. Potential risk factors for development of implant-associated infections during follow-up were investigated with logistic regression models.

**Results:**

A total of 220 patients were included (46.8% males; median age at surgery 46.5 years [IQR: 22–67]; *n* = 23 with implant-associated infections during follow-up). Factors independently associated with subsequent implant-associated infections were smoking (*p* = 0.001), BMI (*p* = 0.015) and radiotherapy (*p* = 0.013). Gender, age, preoperative chemotherapy, allograft material, preoperative C-reactive protein, hemoglobin and reason for surgery (tumor, *n* = 104; trauma, *n* = 13; revision, *n* = 103) were not associated with implant associated infection.

**Conclusion:**

Independent risk factors for implant-associated infections following bone allograft surgery included active smoking, high BMI and radiotherapy (in case of tumor). These factors should be taken into consideration when planning bone allograft reconstruction.

**Supplementary Information:**

The online version contains supplementary material available at 10.1007/s00402-026-06431-z.

## Introduction

Surgical reconstruction of bone defects is one of the most demanding challenges in orthopaedic and trauma surgery [1]. These defects may ensue from tumor resection, severe trauma, or revision surgery after failed primary arthroplasty [2, 3]. Traditionally, autologous bone grafts have been used for reconstruction, as they allow for optimal biological integration and bone healing [4]. However, the amount of available donor bone is limited, and harvesting of autologous grafts is potentially associated with local pain, infection, and long-term morbidity at the harvest site [5]. In recent years, the use of allografts, i.e. transplants of donor bone, has become an increasingly attractive alternative [6]. Allografts offer several advantages, such as immediate availability in large quantities, the ability to treat large bone defects, and no donor-site morbidity [7].

Despite these obvious advantages, allografts have some challenges. One of the most serious complications associated with allograft implantation is postoperative implant-associated infections [8]. These can have serious consequences, including impaired graft integration, delayed healing, and, ultimately, reconstruction failure with ensuing further surgical interventions [9]. The pathogenesis of these infections is complex and influenced by a variety of factors, including the patient’s immune response, the quality of the allograft, and intraoperative contamination [9, 10].

Given the increasing importance of allografts in clinical practice, a thorough understanding of the incidence and risk factors of postoperative implant-associated infections is crucial. However, existing evidence on such infections following allograft implantation remains limited and heterogeneous, underscoring the need for systematic investigation in this field.

Therefore, the present study aims to systematically evaluate the incidence and identify potential risk factors for implant-associated infections after bone allograft implantation. By providing comprehensive data, this study seeks to enhance current knowledge and inform strategies to prevent infection-related complications in reconstructive bone surgery.

## Methods

This retrospective analysis was approved by the local research ethics committee (ECS Graz Nr: 1205/2024). Data of all patients who underwent surgery with a bony allograft at a single university hospital between January 1st, 2003 and December 31st, 2023 was collected. Patients who had received a bone allograft outside of the study period or had a history of infection at the donor site prior to allograft implantation were excluded. This study is reported according to the STROBE guidelines.

The indication for the allograft used was investigated, i.e. whether it was used for bone defect reconstruction following tumor resection, trauma, or orthopaedic revision procedure. Further, the type of allograft (cancellous bone material/allograft struts) was documented. Comorbidities (diabetes mellitus (either type 1 or 2), arterial hypertension, body mass index [BMI] [11, 12], chronic obstructive pulmonary disease [COPD], coronary heart disease, smoking status [active smoker/non-smoker]), the preoperative American Society of Anesthesiologists Classification (ASA) score, and preoperative laboratory parameters (hemoglobin, C-reactive protein [CRP]) were ascertained [13, 14].

Infection was defined using a composite definition adapted from the Musculoskeletal Infection Society (MSIS) criteria and the Centers for Disease Control and Prevention (CDC/NHSN) surgical site infection definitions [15, 16]. 

Specifically, infection required either:

1. The presence of a sinus tract communicating with the implant,
2. Purulence around the graft, or.
3. Isolation of a pathogen from ≥ 2 separate deep tissue or implant samples,
4. Combined with clinical signs of infection and/or elevated inflammatory markers (CRP, WBC).

Cases not fulfilling these criteria were not classified as infections.

Separate analyses were performed for the use of cancellous bone allograft or solid bone allograft. These 2 groups were also analyzed separately.

All allografts used in this study were obtained from the tissue bank of the local orthopaedic center. The bone allografts were harvested from deceased donors, and were processed without irradiation. They were preserved as fresh-frozen grafts at temperatures below − 50 °C. The tissue bank is subject to rigorous quality control and is certified biannually by the government-regulated Agency for Health and Food Safety, ensuring compliance with national and international standards for tissue procurement, processing, and storage.

### Statistics

Data are presented as mean with standard deviations (SD) or median and interquartile range (IQR) as appropriate. The distribution of continuous variables was checked with the Shapiro-Wilk test. Chi-square tests, Fisher’s exact tests, T-tests and Wilcoxon rank-sum tests were used to detect differences between groups followed by post-hoc tests with Bonferroni adjustment, as appropriate. The time from surgery using the allograft to the occurrence of an implant-associated infection was documented, as well as the time from surgery using the allograft to last follow-up or death. Factors potentially associated with the development of implant-associated infections were investigated with logistic regression models. A p-value < 0.05 was considered statistically significant.

## Results

We included 220 patients in the final analysis. The median age at time of allograft implantation was 46.5 years [Interquartile Range (IQR) 22–67 years]. 46.8% of patients were male. Twenty-three (10.5%) developed an implant-associated infection and 197 (89.5%) did not. There was no significant difference in gender or age distribution between the two groups (Table 1).

**Table 1:** Baseline characteristics of the groups

	Implant-associated infection		
	Yes (*n* = 23)	No (*n* = 197)	*P* Value
Age, years, mean (IQR)	51.9 (24.1, 67.9)	45.9 (20.2, 64.8)	0.347
Gender, male (%)	13 (57%)	90 (46%)	0.324
Primary Diagnosis Tumor Trauma Orthopaedic revision	8 (34.8%) 2 (8.7%) 13 (56.5%)	96 (48.7%) 11 (5.6%) 90 (45.7%)	0.427
BMI, mean (IQR)	29.1 (23.0, 32.7)	24.7 (21.3, 27.8)	0.004
Karnofsky, mean (IQR)	94.8 (100, 100)	95.2 (100, 100)	0.670
Cancellous allograft material, n (%)	7 (30)	90 (47)	0.346
Strut allograft, n (%)	16 (70)	107 (53)	0.147
Smoker, n (%)	12 (52)	39 (20)	< 0.001
Hypertension, n (%)	11 (47)	69 (35)	0.227
Diabetes, n (%)	5 (22)	13 (7)	0.013
COPD, n (%)	2 (3)	5 (3)	0.113
Heart failure, n (%)	6 (26)	36 (18)	0.367
Preoperative C-reactive protein (mg/dL), mean (IQR)	6.6 (2.5, 8.2)	7.0 (1.2, 8.1)	0.020
Preoperative hemoglobin, mean (IQR)	13.3 (11.3, 14.9)	13.3 (12.2, 14.2)	0.914
ASA, n (%) I II III IV Time to infection after surgery in days, mean (IQR)	4 (5) 6 (8) 10 (19) 3 (30) 170 (67, 408)	73 (95) 73 (92) 44 (81) 7 (70)	0.014*

P-value based on Fisher’s exact test

*pairwise comparisons with Bonferroni adjustment did not yield statistically significant differences between groups

One-hundred-four patients had received the bone allograft for a defect ensuing tumor surgery (47.3%), 13 (5.9%) following major trauma, and 103 (46.8%) during orthopaedic revision surgery. Of these, 7.7% (8/104), 15.4% (2/13) and 12.6% (13/103) developed implant-associated infections. The infections occurred at a median of 170 days (IQR 67–408) after surgery (Table 1).

In 89.5% (*n* = 197) of patients, laboratory parameters were obtained 1 day preoperatively or at day of surgery. 12 Tumor patients received Radiotherapy, of these 50% (*n* = 6) received it preoperatively. Chemotherapy was used in 22% (*n* = 23) preoperatively. In 5.0% (*n* = 11) of patients, laboratory values were measured 2 days preoperatively, and in 5.5% (*n* = 12) more than 2 days preoperatively.

High BMI (*p* = 0.004), diabetes (*p* = 0.013), high ASA score (*p* = 0.014; significance lost after pairwise comparison with Bonferroni adjustment), elevated preoperative CRP (*p* = 0.020) and active smoking (*p* < 0.001) were significantly associated with the development of implant-associated infections in the univariate analysis. However, we must note that CRP, diabetes, and the ASA score are not independently associated with allograft infection, as well as the remaining risk factors (Table 1**)**. In patients with a tumor diagnosis as the reason for allograft implantation, none of the parameters investigated was significantly associated with altered risk for implant-associated infection in the univariate analysis. (Table 2).

**Table 2:** Univariate analysis of risk factors associated with development of implant-associated infections in patients with a tumor diagnosis

	Implant-associated infection		
	Yes (*n* = 8)	No (*n* = 96)	*P* Value
Presence of distant metastases, n (%)	1 (13%)	22 (23%)	0.121
Resection margin R0, n (%)	8 (100%)	95 (99%)	0.793
Chemotherapy, n (%)	3 (28%)	20 (21%)	0.176
Radiotherapy, n (%)	3 (38%)	9 (9%)	0.090

**Fig. 1:**
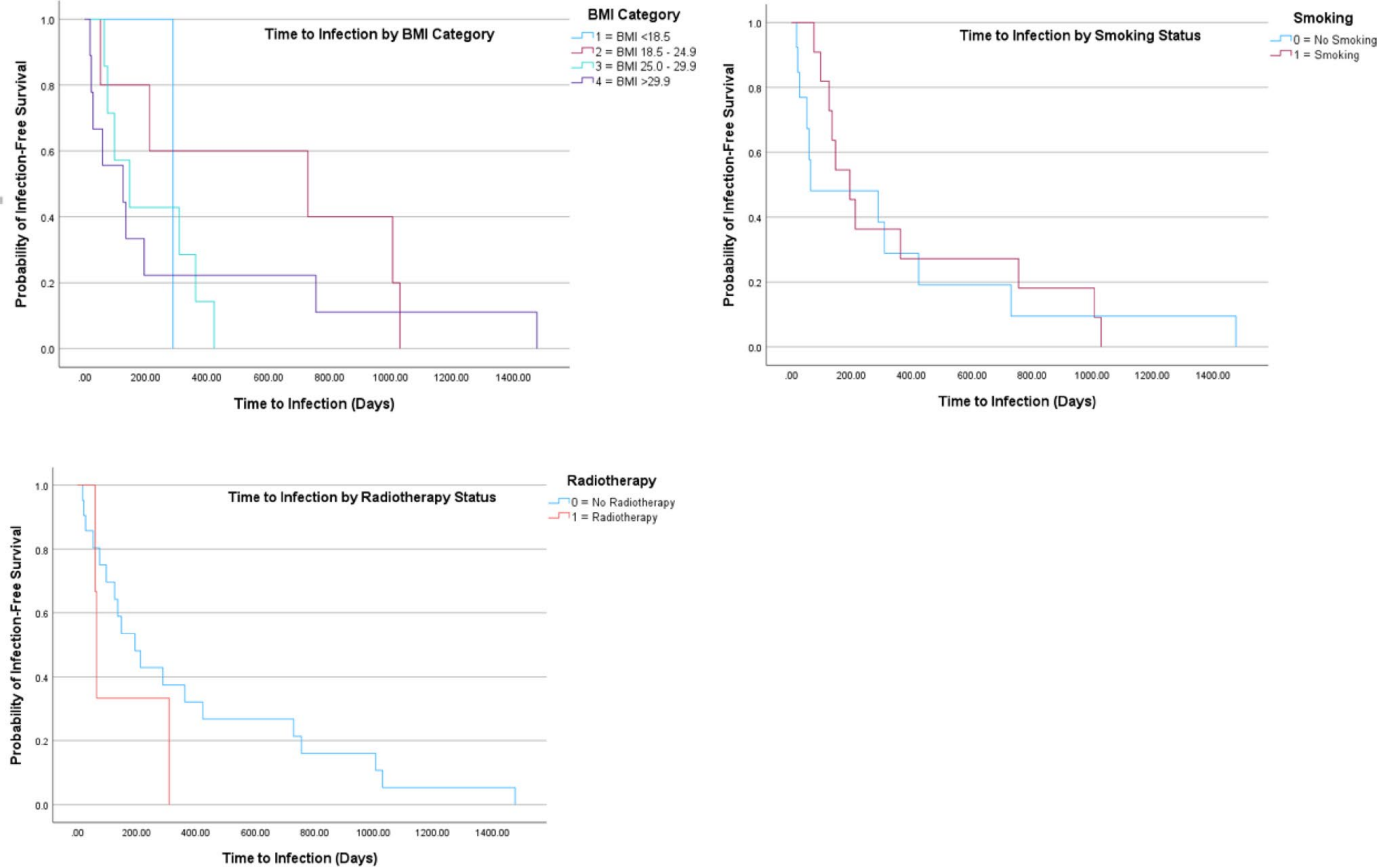
Kaplan-Meier estimates of infection-free survival by BMI category, smoking status and radiotherapy

According to the multivariate logistic regression model, high BMI (*p* = 0.015), pre- or postoperative radiotherapy (*p* = 0.013), and smoking (*p* = 0.003) were independently associated with higher risk for implant-associated infections. (Table 3; Fig. 1).

**Table 3:** Multivariate logistic regression model investigating the independent association of risk factors with development of implant-associated infections

BMI (continuous)	Odds Ratio	95% Confidence Interval	*P* Value
	1.11	1.04–1.21	0.015
Radiotherapy (yes vs. no)	2.51	1.01–6.26	0.013
Smoker (active vs. non-smoker)	3.49	1.37–8.87	0.003

### Treatment of implant-associated infections

All 23 patients with allograft infection underwent at least one revision graft surgery. They received specific antibiotic therapy depending on the pathogen. Four patients (17%) with infection eventually had a major limb amputation, while there was one amputation in the group without infection (1%). In addition, a detailed list of all pathogens found in postoperative allograft infections and their frequency has been compiled for a better overview in the supplementary table. (Table S1)

## Discussion

Across all patients and indications, the postoperative implant-associated infection rate following bone allograft implantation for an osseous defect was 10.5%. Similar values have been reported in other studies, ranging between 2.6% and 18% [17–20]. The primary diagnosis showed a tendency in the infection rate. However it was not significantly lower in tumor disease (8%) than in orthopaedic revision surgery (13%) or major trauma with osseous defect (15%). A large study by Aponte Tinao et al., including more than 400 tumor surgeries using bone allograft and a minimum follow-up of 2 years, reported on an infection rate of 9% [21], similar to the herein discovered 8% for tumor patients. On the other hand, following orthopaedic revision surgery, an infection rate of even 21%, based on 24 procedures with at least 1 year of follow-up has been reported [22].

We also evaluated preoperative laboratory parameters as potential risk factors. Elevated preoperative CRP was significantly associated with postoperative infection in the univariate analysis. However, due to the limited number of infection events, CRP was not included in the multivariate analysis, and its independent predictive value could not be assessed.

As shown in previous studies in various studies of surgical procedures, our study is also consistent with the finding that smoking and an increased BMI are independently associated with an increased risk of postoperative infection in general [23, 24]. Higher ASA classification was significantly associated with implant-associated infection in the univariate analysis. However, due to the limited number of infection events, ASA classification was not included in the final multivariate model, and its independent association with postoperative infection could not be confirmed. Nevertheless, it is known that higher ASA classification is associated with increased risk for surgical site infections [25, 26].

In 2014, Gradl et al. showed in over 1500 tumor orthopaedic surgeries that a high BMI is significantly associated with an increased risk for postoperative infection in patients with preexisting implants [27]. However, the same study also revealed that a primary malignant tumor is independently associated with higher postoperative infection risk. In our subgroup, it shows that it does not confer an increased risk. One difference may be that they included all tumor procedures and we only included those with bone graft implantation, which shows that it does not confer an increased risk in our subgroup.

In the subgroup with tumor as the primary diagnosis, the presence of distant metastases and R0 resection were not significantly associated with an increased postoperative infection risk. Pre- or postoperative chemotherapy did not significantly increase the risk of postoperative bone graft infection. Wisnoyotin et al. 2022 showed in almost 100 patients that chemotherapy has an effect on the non-union rate and structural failure of the graft [28]. This is understandable since chemotherapy slows down the local postoperative healing. However, the infection rate was not specifically evaluated in this study.

In our study, radiation therapy had a significant impact on the postoperative infection risk (*p* = 0.013). This is most likely due to the avascular and poorly perfused local tissue in the surgical area [29]. In a similar study of 73 soft tissue sarcomas by Potkrajcic et al. 2022, a significant increase in wound complications was also observed with preoperative radiotherapy, with a median follow-up of 4.83 years. This confirms our hypothesis that this is an independent risk factor [30]. Nevertheless, preoperative radiotherapy has been used in several series to improve the rate of limb-sparing surgery [31, 32].

### Clinical implications

The findings of this study have several important clinical implications. Patients with modifiable risk factors should be optimized preoperatively to reduce the risk of implant-associated infections following allograft implantation.

- Smoking cessation: Given the association between smoking and impaired graft integration, smoking cessation should be strongly encouraged before surgery.
- BMI optimization: Obesity was identified as a potential risk factor; therefore, preoperative weight optimization may lower infection risk.
- Radiotherapy: Patients with prior radiotherapy represent a high-risk cohort and require careful surgical planning and intensified postoperative surveillance.

These measures may help improve surgical outcomes and reduce complication rates after allograft reconstruction.

This study has several limitations that should be acknowledged. First, the retrospective, single-center design may limit the generalizability of the findings and introduce selection bias and confounding factors. Second, the relatively small number of infection events (*n* = 23) restricts the statistical power of the multivariate analysis, especially for subgroup analyses, such as those involving tumor patients (*n* = 8 with infection). This limited event count may also explain why certain factors lost statistical significance after adjustment. Third, the heterogeneity of the patient population, which includes tumor, trauma, and revision cases, reflects real-world clinical practice but introduces variability because the underlying pathophysiology and risk profiles differ across these groups. These complexities should be considered in the discussion when interpreting the results.

## Conclusion

Our study highlights important independent risk factors for postoperative infection after bone allograft surgery. Smoking, increased BMI, and prior radiotherapy significantly contribute to an increased risk of infection, emphasizing the need for thorough preoperative risk assessment. Given the significant impact of radiation therapy on infection risk, careful surgical planning and postoperative management are essential. Future studies with larger cohorts and longer follow-up are needed to further refine risk stratification and optimize patient outcomes.

## Supplementary Information

Below is the link to the electronic supplementary material.

Supplementary Material 1

## Data Availability

No datasets were generated or analysed during the current study.
