# Supplementary material for: Risk factors for implant-associated infection after bone allograft reconstruction: a 20-year retrospective cohort study
Source: Arch Orthop Trauma Surg. 2026 Jul 23;146(1):266. doi: 10.1007/s00402-026-06431-z (PMC13395846; doi:10.1007/s00402-026-06431-z)
Supplement: Supplementary file 1 — Supplementary Material 1 [file 402_2026_6431_MOESM1_ESM.docx]

**Table S1** Pathogens

| Pathogens | Patients, n |
| --- | --- |
| Staphylococcus aureus (MSSA) | 6 |
| Staphylococcus epidermidis | 4 |
| Cutibacterium acnes | 2 |
| Enterococcus faecalis | 2 |
| Escherichia coli | 1 |
| Klebsiella pneumoniae | 1 |
| Staphylococcus aureus (MRSA) | 1 |
| Propionibacterium avidum | 1 |
| Pseudomonas stuzeri | 1 |
| Pseudomonas aeruginosa | 1 |
| Staphylococcus capitis | 1 |
| Staphylococcus lugdunensis | 1 |
|  |  |
| Candida albicans | 1 |
| Candida glabrata | 1 |
